# PhytoTypeDB: a database of plant protein inter-cultivar variability and function

**DOI:** 10.1093/database/bay125

**Published:** 2018-12-14

**Authors:** Marco Necci, Damiano Piovesan, Diego Micheletti, Lisanna Paladin, Alessandro Cestaro, Silvio C E Tosatto

**Affiliations:** 1 Department of Biomedical Sciences, University of Padua, via U. Bassi 58/b, Padua, Italy; 2 Department of Agricultural Sciences, University of Udine, via Palladio 8, Udine, Italy; 3 Fondazione Edmund Mach, Via E. Mach 1, 38010 S. Michele all'Adige, Italy; 4 Consiglio Nazionale delle Ricerche Institute of Neuroscience, via U. Bassi 58/b, Padua, Italy

## Abstract

Despite a fast-growing number of available plant genomes, available computational resources are poorly integrated and provide only limited access to the underlying data. Most existing databases focus on DNA/RNA data or specific gene families, with less emphasis on protein structure, function and variability. In particular, despite the economic importance of many plant accessions, there are no straightforward ways to retrieve or visualize information on their differences. To fill this gap, we developed PhytoTypeDB (http://phytotypedb.bio.unipd.it/), a scalable database containing plant protein annotations and genetic variants from resequencing of different accessions. The database content is generated by an integrated pipeline, exploiting state-of-the-art methods for protein characterization requiring only the proteome reference sequence and variant calling files. Protein names for unknown proteins are inferred by homology for over 95% of the entries. Single-nucleotide variants are visualized along with protein annotation in a user-friendly web interface. The server offers an effective querying system, which allows to compare variability among different species and accessions, to generate custom data sets based on shared functional features or to perform sequence searches. A documented set of exposed RESTful endpoints make the data accessible programmatically by third-party clients.

## Introduction

The quick drop in the cost of new generation sequencing experiments has resulted in many new plant genomes being published. Not all these experiments are later followed by extensive analyses, and genomes often sit untouched after their initial publication (4). Plant genome data are in principle available through core data resources such as Ensembl Genomes (9) and UniProt (20). However, plant-specific data is less integrated than human data and plant-specific databases have also been developed over the years. These range from single species such as *Arabidopsis thaliana* (10) or maize (1) to groups of sequenced genomes, reviewed, e.g. in (16, 17). Plant-specific databases contain genomics data for single species, focusing on open reading frames sequences, expression data, long non-coding RNA, introns/exons, small RNA, expressed sequence tags, quantitative traits loci, phylogeny or chromosome maps. Comparative genomics resources are also available, e.g. PLAZA (21) or Phytozome (7). Functional annotations in plant-specific databases focus on pathways analysis, domains, evolutionary relationships and gene ontology (GO) terms. Additional databases are dedicated to very specific genes/features such as resistance genes in PRGdb (13), phosphorylation sites in PlantsP/PlantsT (18) or plant protease inhibitors in Plant Protease Inhibitors (PLANT-PIs) (5).

Beyond the increase in number of sequenced plant genome, cheap sequencing technologies also facilitated genotyping experiments, resulting in several whole-genome resequencing initiatives mapping different accessions. International efforts to characterize the whole plant variability like the 1001 Arabidopsis genomes (http://1001genomes.org/) (23) or the 3000 rice genomes (http://iric.irri.org/resources/3000-genomes-project) (24). Beyond the project web pages, this information is only partially available in specialized variation databases such as the European Variation Archive (EVA; https://www.ebi.ac.uk/eva/). From those specialized sources, it is not easy to combine the information of genetic variation with functional annotation or to compare variations among different plants. Despite the scientific and economic importance of several crops, existing resources do not have a user-friendly way to encode and organize that information about annotated genetic variants in different accessions. As an example, *Malus x domestica* (domesticated apple) is one of the most economically important fruit crops in the world and it has thousands of known accessions (11), at least 78 of which have been resequenced. However, there is currently no way for researchers to retrieve or visualize information about the differences among these cultivars. Furthermore, none of these resources contains detailed information about the protein complement, its structure or function. To fill this gap left by existing resources, we developed PhytoTypeDB, a database containing the inter-cultivar variability of functionally annotated plant proteins. To date, four different species (*M. x domestica*, *A. thaliana*, *Theobroma cacao* and *Oryza sativa*) are included in PhytoTypeDB as a proof of principle. The modular analysis framework will be applied to additional plant genomes as a continuing effort for data integration.

### Implementation

PhytoTypeDB contains for each species the amino acid sequence with the relative structural/functional annotations of the reference genome (proteome) and all the single-nucleotide variants (SNVs) from resequenced accessions (e.g. cultivars). Data is obtained from publicly available projects or consortia that produce high-density variability annotation, i.e. the *Malus x domestica* resequencing project (2), 1001 Arabidopsis genomes (23) and 3000 rice genomes (24). Each SNV is identified by type (synonymous/non-synonymous/stop-codon) and position along the reference gene sequence. In the web pages, variants from different accessions are shown on the reference gene. Both the reference proteomes and the variants come from publicly available databases, with the exception of the *M. x domestica* where genes were predicted using an in-house pipeline. All reference proteins are annotated with homology relationships and structural/functional features. Homologs are retrieved based on sequence similarity obtained from running Basic Local Alignment Search Tool (BLAST) against UniProt, UniRef50 and UniRef90 (20) to increase search sensitivity while minimizing redundancy. The fast estimator of secondary structure (FESS) is used to produce secondary structure predictions (14). InterProScan (6) and its MobiDB-lite component (12) are used for the detection of sequence `signatures’ (domains, motifs, sites, etc.) and intrinsically disordered regions, respectively. These annotations are used for function prediction using a new version of the Interaction Network GO Annotator (INGA) method (15) to produce GO term (19) predictions. INGA was top ranking in the latest Critical Assessment of Function Annotation experiment and best scoring in the plant cellular component section (8). The production, management and organization of annotations for insertion in the database are handled by an automated in-house Python pipeline. The server back-end relies on the Node.js and MongoDB technologies and exposes RESTful services to access PhytoTypeDB data programmatically by third-party clients. The front-end exploits the Angular and Bootstrap frameworks to make the entire website reactive and responsive, allowing visualization from any device type. Entry pages include a fully dynamic and expandable feature viewer showing sequence annotation supporting the generation of images for publication.

**Figure 1:**
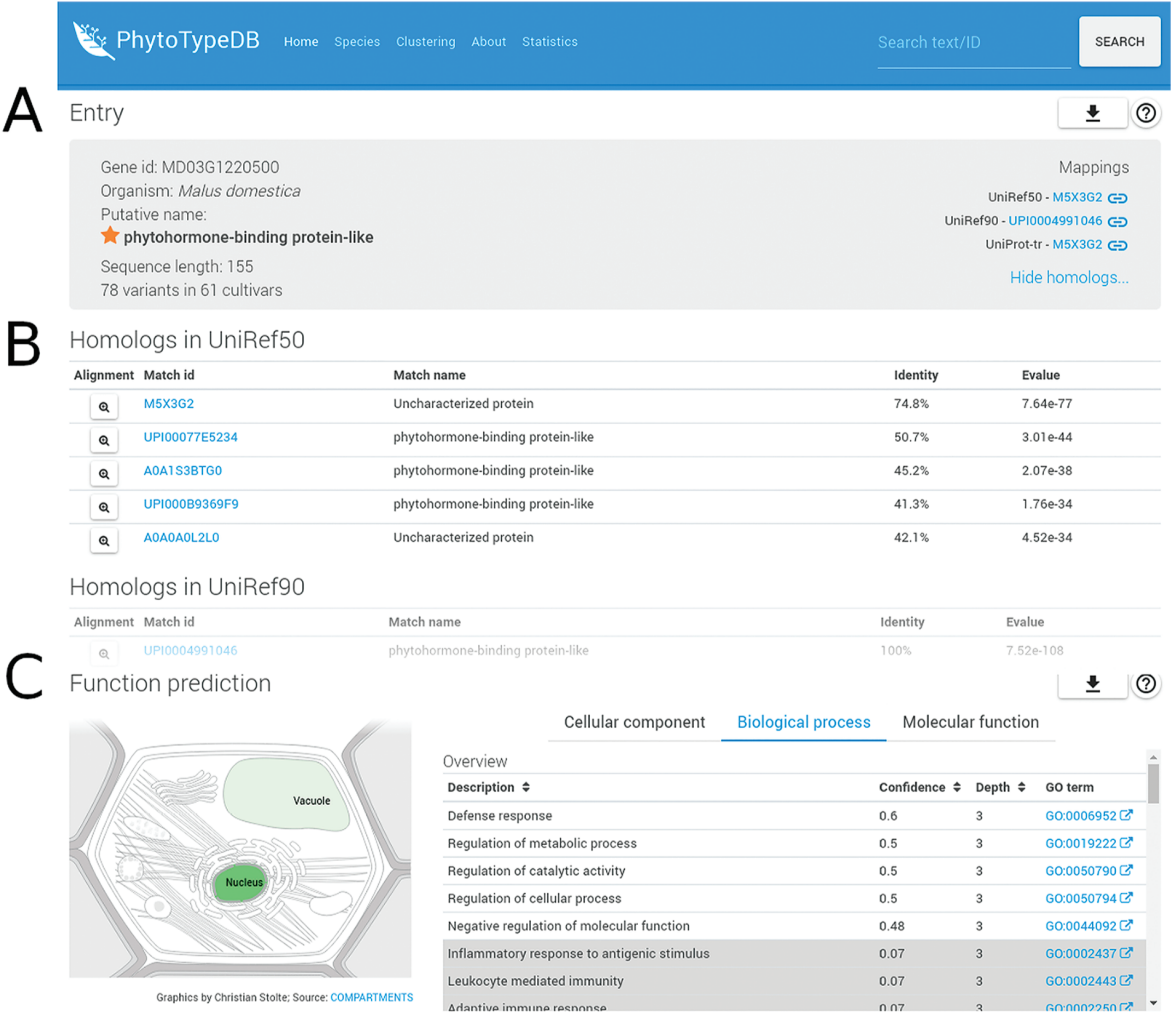
Entry overview. The overview description for PhytoTypeDB entry MD03G1220500 is shown. (**A**) Basic entry information and mappings to UniRef sequence clusters. The gene intrinsic disorder (ID), organism putative name, sequence length and number of variants for different cultivars are shown on the left. Mappings to UniRef sequence clusters are shown on the right and cross-linked to relevant PhytoTypeDB sequence cluster pages. (**B**) Detailed list of homologs found using BLAST. Matching sequences are cross-linked from their ID and corresponding alignments can be visualized by clicking on the magnifying glass icon. (**C**) Function prediction. The left panel graphically shows the sub-cellular compartments predicted by INGA. Three tabs on the right allow the user to switch between GO biological process, molecular function and cellular component. The GO term description is shown alongside its INGA confidence score and a GO term ID cross-linked to the GO website. Low confidence predictions are highlighted with a grey background.

**Figure 2:**
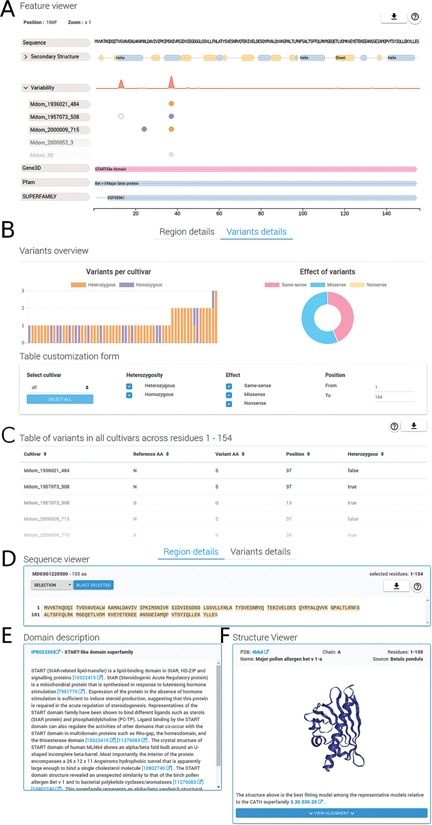
Region and variants description. The detailed region and variants description for PhytoTypeDB entry MD03G1220500 is shown. (**A**) Feature viewer. The sequence feature viewer is fully interactive, allowing to zoom and to select regions of interest; showing sequence, secondary structure, variability and domain annotations. More details can be visualized for secondary structure (helix, strand, coil propensity) and variability tabs (open in the figure) by clicking on their respective tabs. Different cultivars are shown, one per row, with variant sites indicated by empty (same-sense) or full circles (missense) of different colors (purple for heterozygous and gold for homozygous). (**B**) Variants overview showing a histogram for the distribution of variants for each different cultivar. The effect of variants is shown as a pie chart on the right side. A form allows to customize the variants to be shown by selecting cultivars, type of variant, effect and limit positions along the sequence. (**C**) Variants for the selected sequence region are shown in a detailed table containing the cultivar name, reference and variant amino acid type, position and zygosity information. (**D**) Sequence viewer showing the sequence region selected in the feature viewer. Sequence searches using BLAST from the selected sequence can be performed by clicking on the corresponding button. (**E**) A domain description from InterPro is shown after it is selected in the feature viewer. (**F**) Whenever a GENE3D domain is selected in the feature viewer, a separate and interactive structure viewer shows the PDB file with the most similar structure. Details about the PDB file are shown and the sequence to structure alignment can be visualized by clicking on the corresponding button.

### Usage

**Figure 3:**
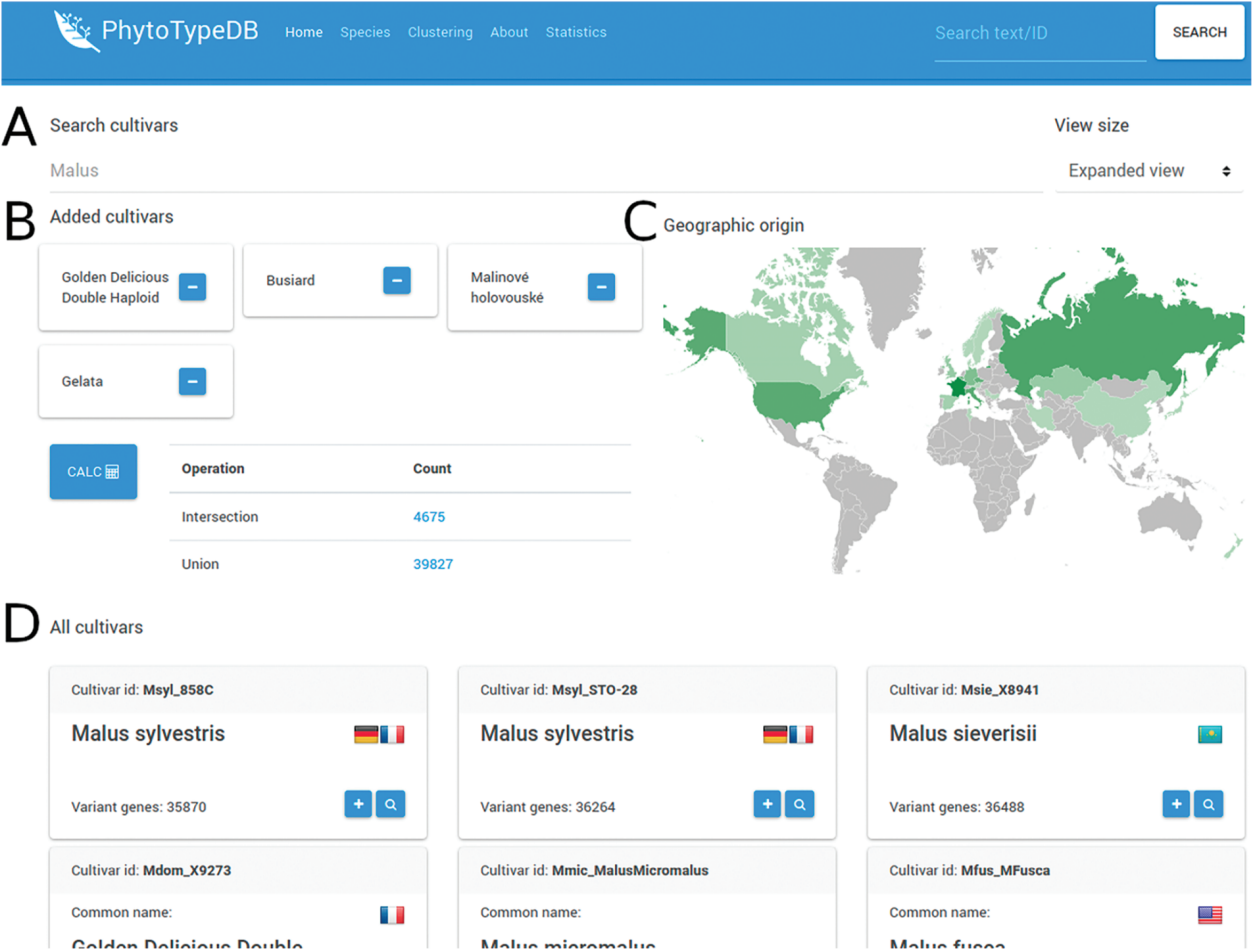
Cultivar selection page. The PhytoTypeDB cultivar selection page is shown for *M. x domestica* . (**A**) Cultivars can be selected directly by their name where known. (**B**) Selected cultivars can be joined into complex searches for the intersection and union of genes with variants. The calculation is made on the fly after clicking the button, updating the statistics. Clicking on the count column will lead to a separate search page where the matching sequence entries are listed. (**C**) The geographic origin of the cultivars visualizes the countries of origin of the selected cultivars, with shades of green defining the relative abundance. (**D**) Detailed names of the cultivars containing the ID, full name, country of origin (by national flag) and number of genes with variants relative to the reference genome. The plus button allows to add the cultivar to the selection, while the magnifying glass leads to a separate search page listing the sequences with variants.

PhytoTypeDB is a database of plant proteins, which includes information on their structure and function focusing on intraspecies variability. Data is organized per species and proteins of each species are structurally and functionally annotated, and it is possible to inspect differences and similarities between accessions in a single view. Protein annotations are displayed in the entry page, see, for example, Figures 1–3. Protein names are given from the alignment against UniProt, UniRef50 and UniRef90 sequences and shown on the top view of the entry page together with species of origin, best mappings to sequence databases (UniProt, UniRef50 and UniRef90), sequence length and a summary of the amount of variability along the protein sequence. PhytoTypeDB provides homologs for 96.9% and the 5 best hits of an entry are shown in a table below the entry overview where the alignment can be consulted by opening a dedicated modal. Most genes annotated in the database have no name as they are not present in UniProt or other core data resources. We thus provide a name for 74.8% of the entries by transferring it from the best BLAST hit. GO terms predicted by INGA are displayed in a table alongside the confidence score. Cellular components terms can be visualized on a schematic cell representation with compartment color intensities increasing with confidence score (3).

The region details tab (see Figure 2) will show detailed information about the protein sequence and regions selected on the feature viewer. The region details are divided into the following three sections: the sequence viewer, the domain description and the structure viewer. In the sequence viewer, the protein sequence can be visualized in various color schemes (e.g. Clustal) and sequence regions selected on the feature viewer can be searched with BLAST against the database, enabling the user to search for specific domains. In the domain description section, InterPro regions are provided with an extensive description of their functional role and related literature citations. The structure viewer section will display 3D protein structure model upon selection of a GENE3D domain in the feature viewer. The 3D protein structure models are chosen by highest sequence similarity among the representatives of the CATH domain selected and loaded dynamically from the Protein Data Bank (PDB) (22). Structural related functional details are provided in the ‘Domain description’ box (Figure 2E). Alongside homology information, we currently annotate 86.8% of entries with InterPro ‘signatures’. Structural classification is provided for 53.4% of the entries covering 57.4% of database residues. Intrinsically disordered regions partially complement structural annotations and are found in 41.8% of the entries. All protein entries are provided with secondary structure predictions obtained from FESS. Domain, and secondary structure annotations are visualized in a feature viewer that draws different features covering a sequence. Comparing disorder prediction with the propensity toward helix or sheet conformation helps to identify regions undergoing events of coupled folding and binding. Differences in the sequence between reference genome and cultivar genes are highlighted in multiple ways (Figure 3). Variability hotspots are visualized in the feature viewer near domains and secondary structure predictions. This allows to quickly identify candidate regions influencing the cultivar phenotype. Hotspot visualization can be enlarged to display single-nucleotide polymorphisms (SNVs) with information about their homozygosity and amino acid effect (same-sense/missense/nonsense). SNVs can also be visualized in the variant details tab containing plots in its upper portion and a detailed table below. Plots summarize variant occurrences and amino acid effect, while the table lists all variants along the protein sequence. The table can be customized both through a filtering form and by interacting with the feature viewer, allowing the user to fine-tune what is displayed.

PhytoTypeDB provides a query system oriented at finding specific genes of interest. Genes can be retrieved by gene ID, annotation ID (e.g. GO term, InterPro ID), free text search (putative names, GO, domain text descriptions), cross-reference identifiers assigned by homology (UniProt, UniRef), local sequence similarity (BLAST search) or accession. Research by accession is performed from a dedicated page where accessions for a species are listed (Figure 3). Accessions of interest from the list can be joined for further analysis, returning genes with variants in each (intersection) or any (union) of the selected accessions. The search form on the top of the page will immediately filter the list of accessions and changes will reflect in the geographic distribution of accession origin.

## Conclusions

In conclusion, PhytoTypeDB is a user-friendly resource developed to help plant scientists to retrieve updated information about gene function and variability. It contains data from consortia producing high-density variability annotation in different cultivars and, in the future, new species will be added as more similar projects are published or upon deposition of data that is still not publicly available. Users can browse information either from a gene- or species-centric perspective surfing among the cultivar variations. The most distinctive feature of PhytoTypeDB is the possibility of exploring, at the same time, gene function along with the intraspecific variability. This will become increasingly useful as more resequencing data becomes available. While we are so far limiting our description of variability to SNVs across cultivars/accessions, we aim to add additional annotations in the future, such as structural variation, orphan genes and accession-wise details of gene families, with the final aim of describing the ‘pan-genome’ of each species included in the database.

## Notes

**Database URL:** http://phytotypedb.bio.unipd.it
